# Single-cell transcriptomic analysis reveals heterogeneous features of myeloid-derived suppressor cells in newborns

**DOI:** 10.3389/fimmu.2024.1367230

**Published:** 2024-06-11

**Authors:** Meng Yao, Yingjiao Cao, Juan He, Rui Dong, Gaoyu Liu, Yingying Chen, Jun Wang, Jie Zhou

**Affiliations:** ^1^ Tianjin Institute of Immunology, Key Laboratory of Immune Microenvironment and Disease of the Ministry of Education, Department of Immunology, School of Basic Medical Sciences, Tianjin Medical University, Tianjin, China; ^2^ Department of Immunology, Zhongshan School of Medicine, Sun Yat-sen University, Guangzhou, China; ^3^ Provincial Key Laboratory of Research in Structure Birth Defect Disease and Department of Pediatric Surgery, Guangzhou Women and Children’s Medical Center, Guangzhou Medical University, Guangzhou, China; ^4^ Pediatric Hematology Laboratory, Division of Hematology/Oncology, Department of Pediatrics, The Seventh Affiliated Hospital of Sun Yat-Sen University, Shenzhen, Guangdong, China; ^5^ Department of Clinical Laboratory, The Key Laboratory of Advanced Interdisciplinary Studies Center, The First Affiliated Hospital of Guangzhou Medical University, National Center for Respiratory Medicine, National Clinical Research Center for Respiratory Disease, Guangzhou, China; ^6^ Precision Research Center for Refractory Diseases, Institute for Clinical Research, Shanghai Key Laboratory of Pancreatic Diseases, Shanghai General Hospital, Shanghai Jiao Tong University School of Medicine, Shanghai, China

**Keywords:** PMN-MDSC, neonatal immunity, preterm infants, heterogeneity, single-cell RNA sequencing

## Abstract

The transitory emergence of myeloid-derived suppressor cells (MDSCs) in infants is important for the homeostasis of the immune system in early life. The composition and functional heterogeneity of MDSCs in newborns remain elusive, hampering the understanding of the importance of MDSCs in neonates. In this study, we unraveled the maturation trajectory of polymorphonuclear (PMN)-MDSCs from the peripheral blood of human newborns by performing single-cell RNA sequencing. Results indicated that neonatal PMN-MDSCs differentiated from self-renewal progenitors, antimicrobial PMN-MDSCs, and immunosuppressive PMN-MDSCs to late PMN-MDSCs with reduced antimicrobial capacity. We also established a simple framework to distinguish these distinct stages by CD177 and CXCR2. Importantly, preterm newborns displayed a reduced abundance of classical PMN-MDSCs but increased late PMN-MDSCs, consistent with their higher susceptibility to infections and inflammation. Furthermore, newborn PMN-MDSCs were distinct from those from cancer patients, which displayed minimum expression of genes about antimicrobial capacity. This study indicates that the heterogeneity of PMN-MDSCs is associated with the maturity of human newborns.

## 1 Introduction

The immune system undergoes a dynamic developmental process during the neonatal period for adaptation to environmental challenges, which is essential for newborn health. Exposure to a variety of antigens, such as conolized microbiota, nutritional antigens, and potential pathogens, affects the development of the postnatal immune system development (1). To avoid overactivation of the immune system and hyperinflammation of unharmful foreign antigens, the immune system of infants remains relatively tolerant. There is a peak of regulatory immune cell development in the perinatal stage, which contributes to the control of inflammation immediately after birth (2). Meanwhile, the functionality of neonatal immune cells differs from that of adults. For instance, macrophages from newborns and adults secrete distinct cytokines when subjected to the same stimulus (3). Consequently, investigation of the characteristics of the neonatal immune system is essential for the understanding of infections and inflammatory disorders in infants.

Myeloid-derived suppressor cells (MDSCs) are a heterogeneous population of immature myeloid cells that were generated under certain pathological conditions, such as tumors and infections. The expansion of MDSCs under tumors or infections facilitates immunotolerance and therefore prevents the antitumor or anti-infection immune responses (4, 5). Elimination of MDSCs has potential therapeutic value in tumor immunotherapy (6). In humans, MDSCs are characterized as CD11b^+^CD14^−^CD15^+^/CD66b^+^ polymorphonuclear (PMN)-MDSCs and CD11b^+^CD14^+^HLA-DR^−/low^CD15^−^ monocytic (M)-MDSCs, which exert immunosuppressive function using distinct mechanisms (7). In an earlier study, we demonstrated that the transient appearance of MDSCs at the neonatal stage has a protective role in the control of inflammation. The immunosuppressive function of MDSCs gradually declined with age and was nearly absent in healthy adults (8). Both the frequencies and the immunosuppressive function of MDSCs were reduced in preterm infants compared to term infants, which may contribute to their heightened vulnerability to inflammatory diseases such as necrotizing enterocolitis (9). However, the understanding of the heterogeneity of PMN-MDSCs in human newborns remains elusive.

In order to gain deep insights into the molecular characteristics and heterogeneity of MDSCs in human newborns, low-density CD11b^+^HLA-DR^−/low^ immature myeloid cells from the peripheral blood of term infants, preterm infants, and adult control were subjected to single-cell RNA sequencing (scRNA-seq). Results revealed that neonatal PMN-MDSCs were composed of heterogeneous clusters undergoing continuous maturation. These clusters were defined as PMN-MDSC precursors, classical PMN-MDSC, late PMN-MDSC, and mature neutrophils. Consistent with the fact that preterm infants indicate diminished function of MDSCs, late PMN-MDSCs were found to be more abundant in preterm infants and exhibited reduced gene expression about antimicrobial capacities. In addition, neonatal PMN-MDSCs indicated weaker immunosuppressive function but stronger antibacterial capabilities, as compared with PMN-MDSCs from cancer patients.

## 2 Materials and methods

### 2.1 Human samples

Samples of peripheral blood were collected from healthy adults, full-term infants, and preterm infants. Samples were collected from 32 subjects from Guangzhou Women and Children’s Medical Center and the Third Affiliated Hospital of Guangzhou Medical University. The basic characteristics of the samples are included in **Supplementary Table S2**. All subjects were collected within 7 days after birth and screened for serum hepatitis B surface antigen (HBsAg), hepatitis C virus (HCV) antibody, hepatitis D virus (HDV) antigen, HDV antibody, and HIV antibody, and positive individuals were excluded from this study. Preterm infants were defined as those with a gestational age of less than 37 weeks. Full-term infants were defined as those with gestational age between 37 and 42 weeks. Informed consent was obtained from all participants.

### 2.2 Sample preparation for 10× genomic single-cell sequencing

Peripheral blood samples were collected from three full-term, three preterm, and three adult controls. Single-cell suspensions of peripheral blood mononuclear cells (PBMCs) were obtained by centrifuging human blood samples on a Ficoll gradient. Briefly, blood was mixed with an equal volume of 2% FBS in PBS and gently layered on the Ficoll gradient. Cells were centrifuged at 1,000×*g* for 25 min without braking. The cells in the middle layer were then washed once with PBS, and the red cells were removed using ACK lysing buffer and resuspended in 2% FBS in PBS for use. The PBMC suspensions were stained with surface markers at 30 min at 4°C in the dark. The strategy for MDSCs was CD11b^+^HLA-DR^−/lo^. Live immature myeloid cells in RPMI1640 supplemented with 20% FBS were sorted in a BD FACSAria III cell sorter (BD Biosciences, USA).

### 2.3 Flow cytometric analysis

Freshly prepared PBMCs were incubated with Fc-block (Miltenyi, Bergisch Gladbach, Nordrhein-Westfalen, Germany) for 10 min, and surface staining was performed at 4°C for 30 min. Cells were stained with surface antibodies against CD11b, HLA-DR, CD14, CD177, and CXCR2. For Arg1 staining, single-cell suspensions were stained with antibodies to surface antigen, then fixed and permeabilized, followed by staining with anti-Arg1 (A1exF5; Thermo Fisher, Waltham, Massachusetts, USA). For ROS staining, single-cell suspensions were stained with antibodies to surface antigen and were evaluated with 20 μM DCFDA (Abcam, Cambridge, UK) staining at 37°C for 30 min. The analysis was performed by BD FACSCanto™ II.

The following antibodies from (BioLegend, San Diego, California, United States) were used: APC antihuman CD11b(ICRF44), Brilliant Violet 605™ antihuman HLA-DR(L243), APC/Cyanine7 antihuman CD14(63D3), PerCP/Cyanine5.5 antihuman CXCR2(5E8/CXCR2), and FITC antihuman CD177(MEM-166).

### 2.4 scRNA-seq data collection and analysis

The data about newborns have been deposited in NCBI’s Gene Expression Omnibus and are accessible through GEO Series accession number GSE253963 (https://www.ncbi.nlm.nih.gov/geo/query/acc.cgi?acc=GSE253963). The raw data were preprocessed using *Cell Ranger* (version 7.0). The R toolkit *Seurat* (version 4.0.1) was used for downstream analysis (10). Cell subpopulations were annotated based on the expression of canonical marker genes. The function of *AddModuleScore* was performed to calculate the scores of molecular signatures in individual cells. The gene sets used for each molecular signature are listed in **Supplementary Table S4**. Genes associated with the enriched pathways identified through GO analysis are listed in **Supplementary Tables S3**, **S4**. The scRNA-seq dataset of tumor MDSCs (GSE163834) was downloaded from the Gene Expression Omnibus GEO database (https://www.ncbi.nlm.nih.gov/gds/) (11).

### 2.5 Pseudo-time developmental trajectory analysis

The R package *monocle2* (version 2.26.0) was used to infer the differentiation path among distinct cell subpopulations (12). Briefly, a *CellDataSet* class was created by using a normalized scRNA-seq dataset. Subsequently, the *differentialGeneTest* function was performed to identify differentially expressed genes (DEGs) with each subpopulation. These DEGs were then used as input genes to perform dimension reduction using the *DDRTree* method. Next, each cell was assigned a pseudo-time order by executing the *orderCell* function. Finally, the pseudo-time trajectory was plotted by performing the function of *plot_cell_trajectory*.

### 2.6 Statistical analysis

All data were derived from at least two independent experiments. Statistical analysis was performed with GraphPad Prism 8.0. Statistical significance was determined by a paired Student’s *t*-test or an unpaired Student’s *t*-test for comparing two groups, and a one-way ANOVA with a Tukey–Kramer multiple comparisons test was performed for comparing three groups within the same experiment. A *p*-value < 0.05 was considered significant, and the results showed the mean ± SEM.

## 3 Results

### 3.1 Maturation trajectory of PMN-MDSCs in the peripheral blood of newborns

**Figure 1:**
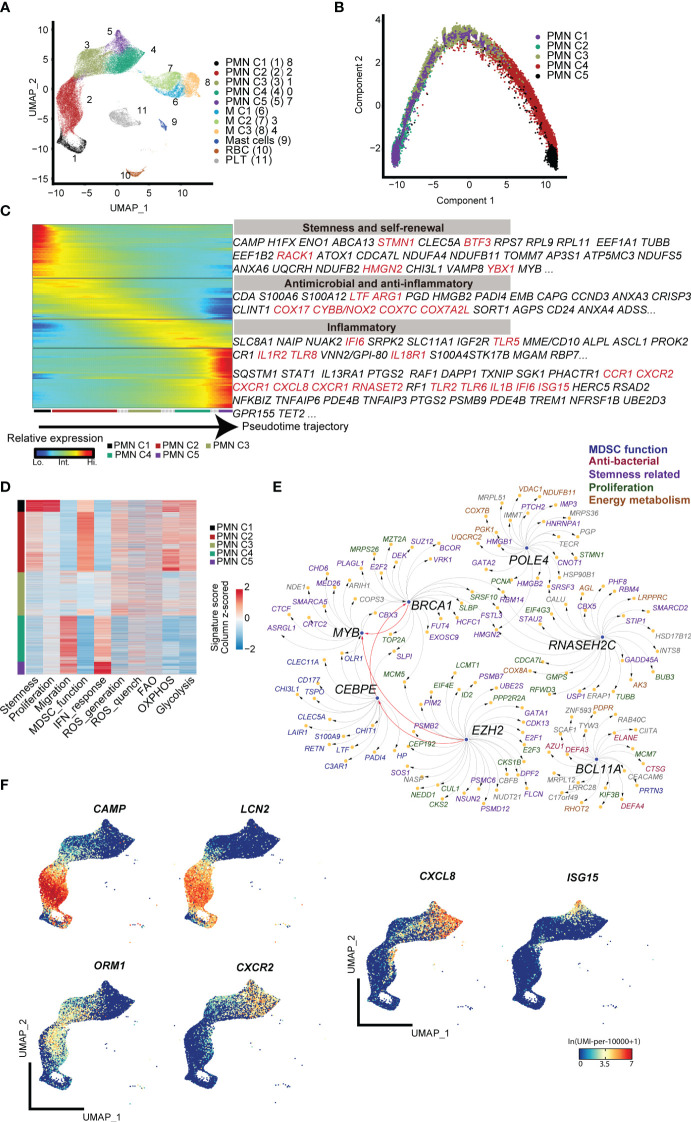
scRNA-seq analysis of steady-state PMN-MDSCs in the peripheral blood of full-term and preterm neonates. **(A)** 2D-UMAP plot showing the distribution of CD11b^+^HLA-DR^-/lo^ MDSCs. **(B)** Scatter plot displaying pseudo-time trajectory that infers developmental orders of PMN-MDSC subpopulations at single-cell resolution. Cell orders are deduced from the expression of the most variable genes across all cells. **(C)** Heatmap displaying expressions of selected marker genes in PMN-MDSCs that are arranged along the pseudo-time trajectory. **(D)** Heatmap displaying normalized scores of molecular signatures across individual PMN-MDSCs. Cells were ordered by their identities. **(E)** Network plot displaying the top transcriptional regulons for PMN C1. **(F)** 2D-UMAP plots visualizing specific gene expressions in PMN-MDSCs at single-cell resolution.

First, low-density CD11b^+^HLA-DR^−/low^ immature myeloid cells from the peripheral blood of term infants, preterm infants, and adult control were subjected to 10× genomic scRNA-seq. Through unsupervised clustering analysis, five subpopulations of polymorphonuclear MDSCs (PMN-MDSCs) and three subpopulations of monocytic MDSCs (M-MDSCs) were identified. Potentially contaminated populations, such as mast cells, red blood cells (RBC), and platelets (PLT), were excluded from the subsequent analysis (**Figure 1A**; **Supplementary Figure S1A**). The PMN-MDSC clusters exhibited a clear developmental trajectory toward maturation and activation (**Figures 1B, C**). Molecular signature analysis revealed that the PMN C1 cluster displayed characteristics of stemness (**Figure 1D**), marked by high levels of genes associated with proliferation (*Mki67*, *Stmn*). Transcriptional factor (TF) analysis indicated that several regulons [*Brca1* (13), *Myb* (14), *Bcl11a* (15), and *Ezh2* (16)] associated with stemness maintenance were active in PMN C1 cells, indicating the self-renewal and pluripotent state of PMN C1 cells (**Supplementary Figure S1A**; **Figure 1E**). The PMN C2 cluster exhibited a typical expression pattern of MDSCs [*Cd66b* (17), *Arg1* (18), and *Ltf* (9)] and high levels of reactive oxygen species (ROS) generation, and highly expressed antibacterial genes (*Hp* (19), *Camp* (20), and *Lcn2* (21) (**Supplementary Figure S1A**; **Figures 1D, F**). Furthermore, PMN C2 highly expressed *Cebpe* (22), a key transcription factor involved in granulopoiesis (**Supplementary Figure S1B**; **Figure 1E**). PMN C3 expressed lower levels of antibacterial genes, but it displayed comparable levels of immunosuppressive genes [*Orm1* (23), *Arg1*, and *Cd177* (24)] as compared with PMN C2 (**Supplementary Figure S1A**; **Figure 1F**). PMN C4 and PMN C5 highly expressed *Cd16b* and migration-related genes (*Cxcr2*, *Cxcl8*, and *Cxcr1*), indicating a mature inflammatory neutrophil phenotype (**Figure 1F**, **Supplementary Figure S1A**). PMN C5 specifically expressed interferon-activated genes such as *Isg20*, *Isg15*, and *Ifit1* (**Figure 1F**; **Supplementary Figure S1A**). Type 1 interferon signaling has been demonstrated to negatively regulate the immunosuppressive function of neonatal MDSCs (25). The MDSC subpopulations from PMN C3 to PMN C5 gradually lost the signature of stemness, immunosuppressive function, and ROS generation while acquiring patterns associated with type I interferon responses and chemotaxis (**Figure 1D**). Based on these characteristics, PMN C1–C3 were classified as precursory-PMN-MDSC, classical-PMN-MDSC, and later-PMN-MDSC, respectively; PMN C4–C5 represented mature neutrophils. In summary, our findings reveal a continuous maturation trajectory of PMN-MDSCs in the peripheral blood of neonates.

Regarding the heterogeneity of M-MDSCs, which were classified into three subpopulations (**Figure 1A**), cluster M1 exhibited increased expression of genes involved in inflammatory response and chemotaxis, such as *Il1b, Tnf*, *Ccl3*, and *Cx3cr1*. Cluster M2 was marked by enhanced expression of *Csf1r* (26), a gene associated with the regulation of hematopoietic precursor cells. Cluster M3 displayed high expression of *Cd163*, which is related to anti-inflammation and tissue repair (**Supplementary Figure S1A**) (27, 28).

### 3.2 Delineating heterogeneity of neonatal PMN-MDSCs by CD177 and CXCR2

In order to find a straightforward flow cytometry-based framework to differentiate the PMN-MDSC clusters, the expression pattern of cell surface markers was analyzed based on scRNA-seq. *Cd177* (24), a glycosyl-phosphatidylinositol (GPI)-linked cell surface glycoprotein, was found to be specifically expressed on PMN C2 and PMN C3 (**Supplementary Figure S1A**). Additionally, *Cxcr2*, which was reported to facilitate MDSC recruitment to tumor, was abundantly expressed in PMN C3–C5 (29, 30) (**Supplementary Figure S1A**). CD177 and CXCR2 were used as potential markers for PMN subsets in a flow cytometry panel.

**Figure 2:**
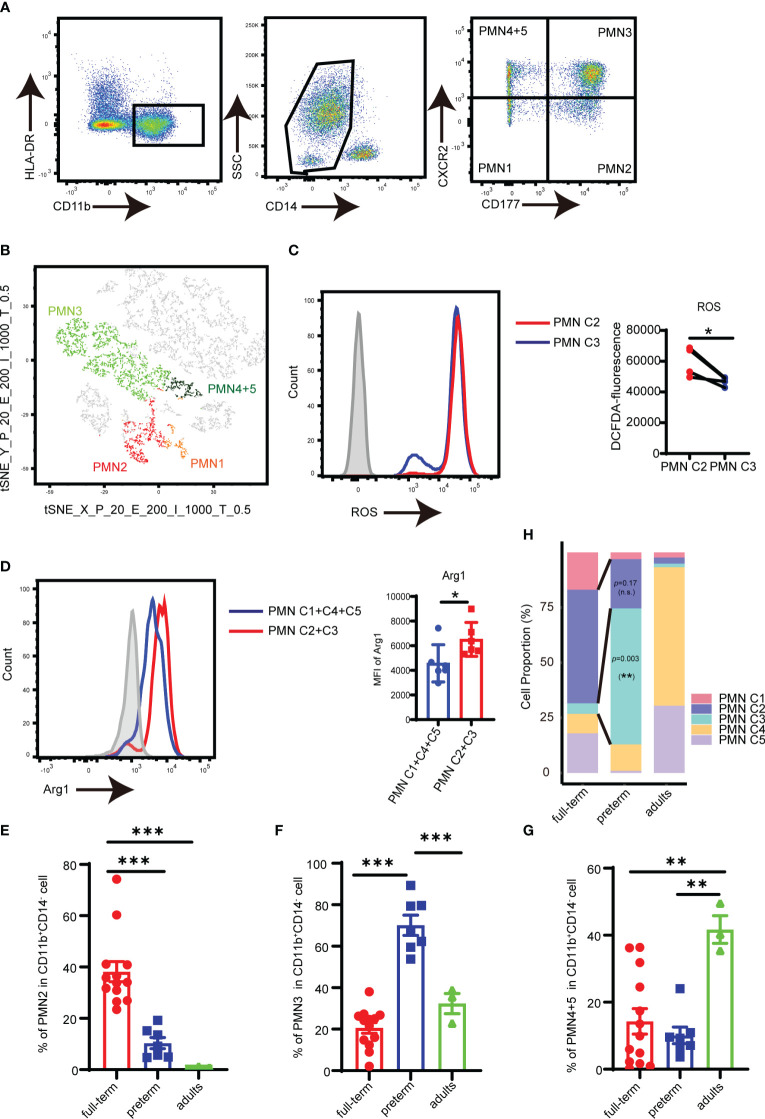
Analysis of PMN-MDSC subpopulations by flow cytometry. **(A)** FACS and staining strategy for PMN C1 (CD177-CXCR2^lo^), PMN C2 (CD177^+^CXCR2^lo^), PMN C3 (CD177^+^CXCR2^hi^), and PMN C4 + 5 (CD177^-^CXCR2^hi^) PMN-MDSC. **(B)** t-SNE of PMN-MDSCs according to the expression of CD177 and CXCR2 by flow cytometry. **(C)** The expression of ROS in PMN C2 and PMN C3 was detected by flow cytometry. **(D)** The expression of Arg1 in PMN C2 and PMN C3 was detected by flow cytometry. **(E)** Percentage of PMN2 in peripheral blood from full-term (*n* = 13), preterm (*n* = 7), and adults (*n* = 3). **(F)** Percentage of PMN4 + 5 in peripheral blood from full-term (*n* = 13), preterm (*n* = 7), and adults (*n* = 3). **(G)** Percentage of PMN4 + 5 in peripheral blood from full-term (*n* = 13), preterm (*n* = 7), and adults (*n* = 3). **(H)** Bar plots comparing frequencies of individual components in the PMN-MDSC compartment among different groups in the scRNA-seq dataset. Blocks represent individual PMN-MDSC subpopulations. The data are representative of two independent experiments. Error bars show mean ± SEM; ^*^ *p* < 0.05; ^**^ *p* < 0.01; ^***^ *p* < 0.001 by two-paired Student’s *t*-test **(C)**, two unpaired Student’s *t*-test **(D)**, or one-way ANOVA with Bonferroni post-test **(E–G)**.

Flow cytometry analysis revealed that PMNs could be divided into four subpopulations based on the quadrants created by CD177/CXCR2: PMN C1 (CD177^−^CXCR2^lo^), PMN C2 (CD177^+^CXCR2^lo^), PMN C3 (CD177^+^CXCR2^hi^), and PMN C4 and PMN 5 (CD177^−^CXCR2^hi^) (**Figure 2A**). T-distributed random neighbor embedding (tSNE) dimensionality reduction was performed on PMNs using flow cytometry. The proximity of the four distinct PMN clusters observed in flow cytometry was similar to the transcriptomic profiling of scRNA sequencing (**Figure 2B**; **Supplementary Figure S2A**). FACS analysis revealed a high level of ROS in PMN C2 (**Figure 2C**) and increased expression of Arg1 in the PMN C2 and PMN C3 cells as compared to PMN C1 and PMN C4 + 5 cells (**Figure 2D**). Meanwhile, PMN C2 was enriched in full-term infants, and the proportion of PMN C3 was higher in preterm infants than in term infants (**Figures 2E, F**). The presence of PMN C3 is strongly negatively correlated (*p* = 0.006) with the birth weight of the infant (**Supplementary Figure S2B**). Conversely, PMN C4 + 5 clusters were predominantly detected in adults (**Figure 2G**). Those results were consistent with the scRNA-seq analysis (**Figure 2H**; **Supplementary Figure S1A**). Thus, a combination of CD177 and CXCR2 could be utilized as a framework to distinguish subsets of neonatal PMN-MDSC.

### 3.3 scRNA-seq shows PMN-MDSCs from preterm neonates indicate diminished functionality

**Figure 3:**
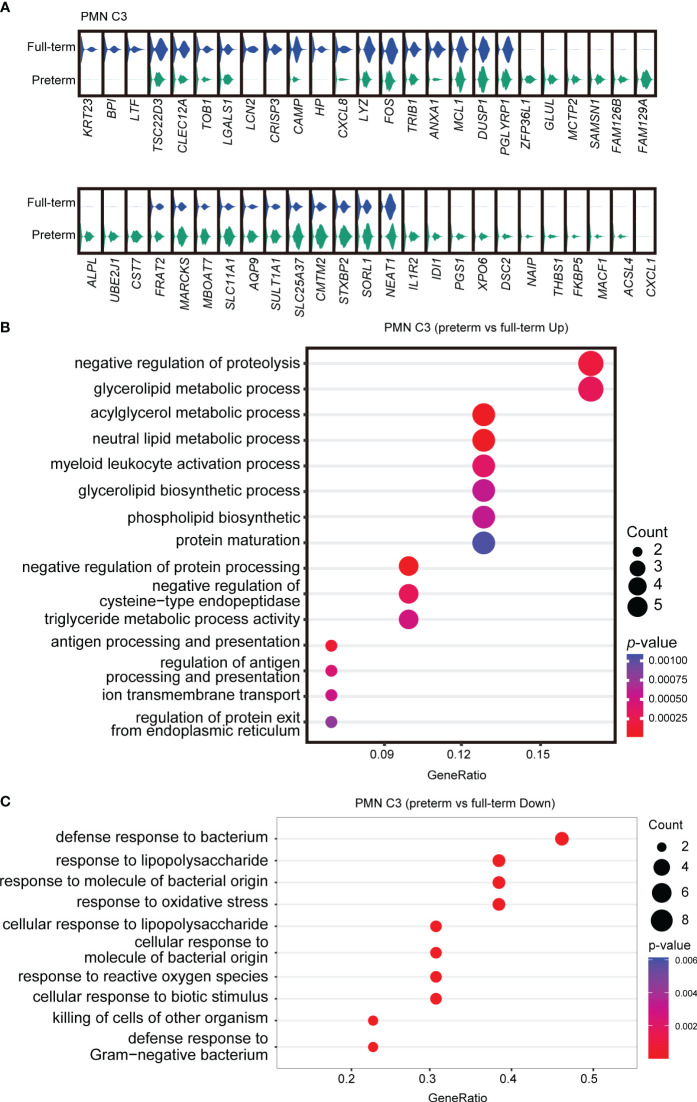
Abnormal functional state of PMN C3 in preterm infants. **(A)** Violin plots comparing gene expressions in PMN C3 between full-term and preterm infants. **(B, C)** Gene ontology enrichment analysis was performed with overexpressed **(B)** or downregulated genes **(C)** in PMN C3 from preterm infants in comparison with full-term infants. Selected GO terms with Benjamini–Hochberg-corrected *p*-values < 0.05 (one-sided Fisher’s exact test) were shown.

To further investigate the functional heterogeneity of PMN-MDSCs in preterm neonates, we analyzed the main clusters (PMN C2 and PMN C3) from preterm neonates compared with those from full-term neonates. PMN C2 exhibited distinct gene expression patterns and molecular signatures when comparing samples from full-term and preterm individuals; the molecular signatures associated with MDSC function were found to be diminished in preterm infants (**Supplementary Figures S3A, B**). The abundance of PMN C3(late PMN-MDSCs) with low expression of genes about antimicrobial capacities significantly increased in preterm neonates compared to full-term neonates (**Figures 2E, H**). Differential expression analysis revealed that PMN C3 from preterm neonates had an overexpression of genes involved in neutrophil maturation and activation [*Cst7* (31), *Marcks* (32), *Stxbp2* (33), *Naip* (34), and *Acsl4* (35)] in comparison to those from full-term newborns (**Figure 3A**). Gene ontology enrichment analysis identified myeloid leukocyte activation and neutral lipid/phospholipid metabolic processes as relevant gene ontology terms (**Figure 3B**). Meanwhile, PMN C3 from preterm neonates exhibited a decreased expression of genes involved in immunosuppression [*Ltf* (36), *Tsc22d3* (37), *Clec12a* (38), *Lgals1* (39), *Lcn2* (40), and *Anxa1* (41)], neutrophil program restraining [*Trib1* (42)], and antimicrobial activity [*Camp*, *Lyz*, and *Bpi* (43)] (**Figure 3A**). Gene ontology analysis with the under-expressed genes revealed relevant terms such as response to lipopolysaccharide and response to oxidative stress (**Figure 3C**). The findings suggest that MDSCs in preterm infants primarily consist of late PMN C3 cells, which subsequently undergo further maturation with compromised immunosuppressive and antimicrobial capacities at the transcriptome level. This may explain the increased occurrence of opportunistic infections among preterm infants.

### 3.4 Neonatal PMN-MDSCs display stronger antimicrobial capacities than tumor PMN-MDSCs by scRNA-seq analysis

**Figure 4:**
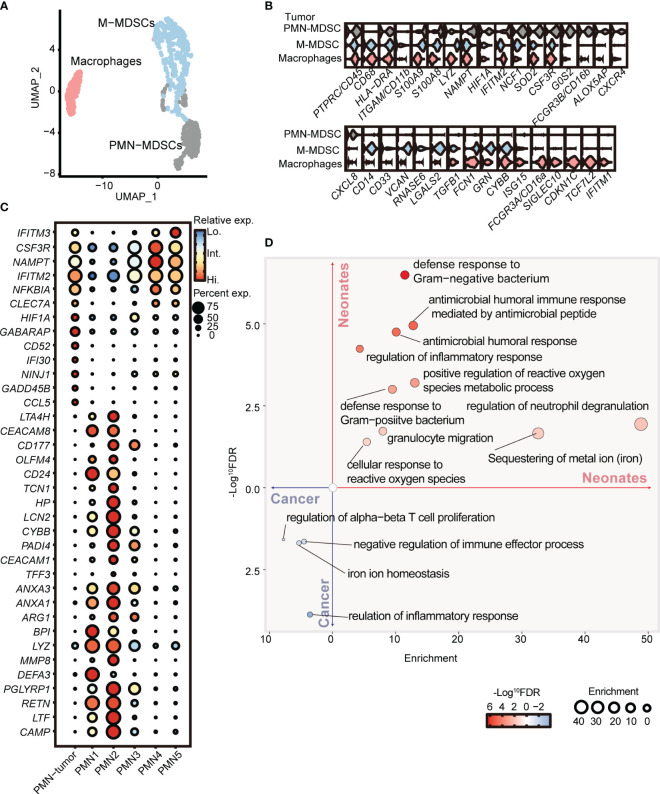
PMN-MDSCs in newborns are distinct from those in cancer patients. **(A)** 2D-UMAP plot showing the distribution of myeloid subpopulations in the peripheral blood of cancer patients. **(B)** Violin plots showing marker gene expressions across PMN-MDSCs, M-MDSCs, and macrophages. **(C)** Dot plots displaying differential gene expressions in PMN from tumor patients and full-term neonates. **(D)** GO enrichment analysis was performed with differentially expressed genes between PMNs from cancer patients and full-term neonates.

To further elucidate the distinctions between PMN-MDSCs derived from neonates and cancer patients, we conducted a comparative analysis of our scRNA-seq data obtained from full-term neonates and a previously published dataset of tumor MDSCs (GSE163834) (11). Unsupervised clustering partitioned the myeloid cells from the peripheral blood of cancer patients into PMN-MDSCs, M-MDSCs, and macrophages (**Figures 4A, B**). Notably, PMN-MDSCs obtained from cancer patients exhibited elevated expression levels of genes associated with tumor progression, including *Ninj1* (44), *Hif1a* (45), *Gadd45b* (46), and *Cd52* (47). Interestingly, the expression levels of *Ifitm2* and *Ifitm3*, pivotal players in cellular antiviral defense (48), were elevated in PMN-MDSCs derived from cancer patients. Furthermore, the overexpression of *Ifitm2* and *Ifitm3* within the tumor cell contributes to tumor progression and metastasis (49–51). Conversely, PMN C2 from neonates displayed overexpression of *Pglyrp1* (52), *Hp*, and *Lcn2*, genes known to be involved in antimicrobial infection (**Figure 4C**). Gene ontology enrichment analysis revealed that PMN C2 from neonates exhibited overexpression of genes associated with heightened antimicrobial humoral response and positive regulation of the reactive oxygen species metabolic process (**Figure 4D**), both of which are closely linked to the antimicrobial functions of MDSCs. In contrast, PMN-MDSCs from cancer patients displayed overexpression of genes enriched in negative regulation of immune effector processes (**Figure 4D**). In conclusion, our findings indicate that PMN-MDSCs derived from neonates not only play a significant role in immunosuppression but also serve as key contributors to antimicrobial infection, which is crucial for newborns with an immature immune system. Conversely, PMN-MDSCs within tumors solely exhibit gene expression profiles about immunosuppressive function to facilitate tumor progression.

## 4 Discussion

Our study identified heterogeneities within the peripheral blood of human neonatal PMN-MDSCs based on the states of neutrophilic maturation. These states can be distinguished by the expression of CD177 and CXCR2. We observed that preterm neonates primarily have late PMN-MDSCs with decreased expression of genes about antimicrobial capacities. Furthermore, we have found that PMN-MDSCs from neonates exhibit potent gene expression of antimicrobial capacity compared to those from tumor patients. Our study provided valuable insights into the characteristics of neonatal MDSCs in the peripheral blood and provided a reference for understanding MDSC function in the treatment of neonatal infections.

The heterogeneities of myeloid-derived suppressor cells (MDSCs) have been documented, and specific markers such as CD84 and CD14 have been identified within tumor microenvironments. Studies reported MDSC differentiation trajectory from neutrophil progenitors through an aberrant path of differentiation in cancer (11, 53). Our results show PMN-MDSC in newborns can cause a loss of immunosuppressive and antimicrobial function with a mature trajectory. The commonly used flow cytometry panel of CD11b^+^CD14^−^CD15^+^/CD66b^+^ is insufficient to accurately differentiate the heterogeneities of neonatal PMN-MDSCs. To overcome this limitation, we have incorporated two additional surface markers (CD177^+^CXCR2^lo^) that precisely capture the major subpopulation among neonatal PMN-MDSCs. The abundance of later PMN C3 (CD177^+^CXCR2^hi^) had a negative correlation with the birth weight of the infants, and the birth weight of infants was associated with the risk of NEC (9). This improvement in detection accuracy will greatly benefit future research in this field and may have prognostic value for neonatal inflammatory diseases.

Preterm infants displayed a heightened proinflammatory cytokine response (54). Neonatal PMN-MDSC treated with lactoferrin (9) and adenosine (55) improved their immunosuppressive and antimicrobial functions and displayed a protective therapeutic effect on necrotizing enterocolitis, which was susceptible in preterm infants. Our findings indicate that PMN-MDSCs from preterm infants exhibit a transcriptomic state of further neutrophilic maturation and decreased antimicrobial capacity. The enrichment of late PMN-MDSCs in preterm infants may be attributed to accelerated maturation. Further studies are required to investigate the possibility of inhibiting the differentiation and maturation of MDSCs in preterm infants and restoring their antimicrobial ability. These findings have important clinical implications for the treatment of preterm neonatal infections.

Results showed that the level of PMN-MDSC in the peripheral blood of cancer patients was significantly higher than that of healthy donors (56). PMN-MDSC exerted immunosuppressive effects by inhibiting the proliferation and function of T cells, depending on the cancer (57). The mechanisms of immunosuppression included: (1) direct contact with T cells by the major histocompatibility complex (MHCII) results in the loss of T-cell response to antigen-specific stimulation; (2) unspecifically inhibited T-cell function by producing Arg1 (58). In recent years, we found that PMN-MDSC showed antimicrobial capacity and killed bacteria in neonates (8, 9, 55). In this study, by comparing PMN-MDSCs from tumors and neonates, we have discovered that the gene expression of the antimicrobial capacity of PMN-MDSCs is specific to the neonatal period.

Despite the limitations of our study, such as the limited availability of neonatal specimens and the inability to perform additional functional validations (the assay of immunosuppressive function and antibacterial activity) for each subpopulation of PMN-MDSCs, we have provided valuable insights into the characteristics and functions of neonatal MDSCs through sc-RNA seq analysis and the protein expression of immunosuppressive molecules. Meanwhile, due to the stringent inclusion criteria and the scarcity of samples from preterm infants, we were unable to acquire a larger sample size within a specified timeframe. Additionally, we were unable to obtain meaningful information from subpopulations of M-MDSCs in neonates due to their low cell proportion and insufficient sequencing depth.

## Data availability statement

The datasets presented in this study can be found in online repositories. The names of the repository/repositories and accession number(s) can be found below: GSE253963 (GEO).

## Ethics statement

The study was approved by the clinical ethics review boards of Guangzhou Women and Children’s Medical Center, the Third Affiliated Hospital of Guangzhou Medical University. All participants, or their legal guardians, signed informed consent forms.

## Author contributions

MY: Writing – original draft. YjC: Writing – review & editing. JH: Conceptualization, Methodology, Writing – review & editing. RD: Conceptualization, Methodology, Writing – review & editing. GL: Resources, Writing – review & editing. YyC: Writing – review & editing. JW: Formal Analysis, Writing – review & editing. JZ: Writing – review & editing.
