# Supplementary material for: Single-cell transcriptomic analysis reveals heterogeneous features of myeloid-derived suppressor cells in newborns
Source: Front Immunol. 2024 Jun 11;15:1367230. doi: 10.3389/fimmu.2024.1367230 (PMC11196393; doi:10.3389/fimmu.2024.1367230)
Supplement: Supplementary file 1 [file DataSheet_1.docx]

Supplementary Material

# Supplementary Figures and Tables

## Supplementary Figures


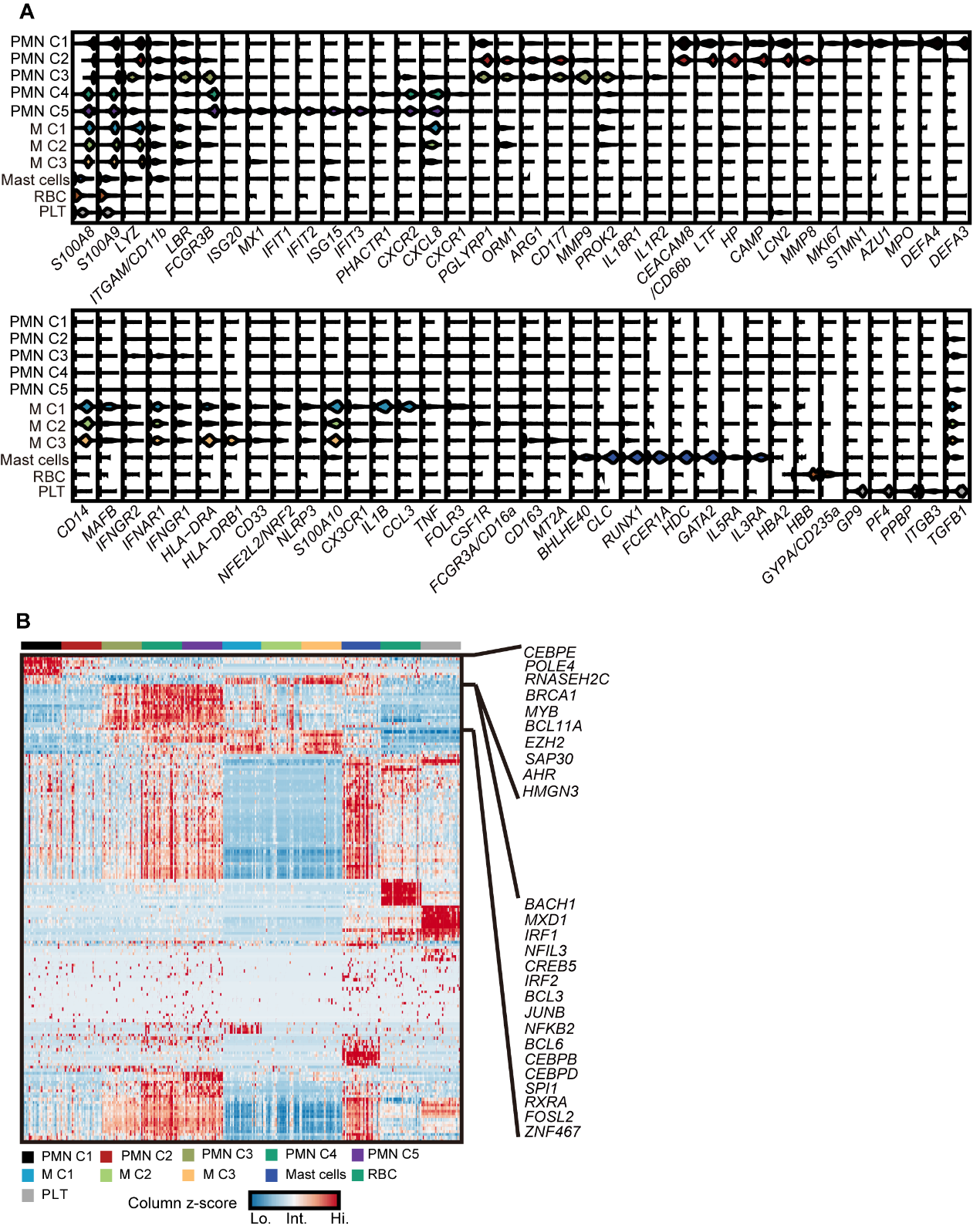


**Supplementary Figure 1.** scRNA-seq analysis of steady-state Full-term, Preterm and Adults MDSCs, related to Figure1. **(﻿A)** Violin plots showing the maker genes of within each PMN-MDSCs and M-MDSCs clusters. **(B)** Heatmap displaying expression of transcription factors within each MDSC clusters.


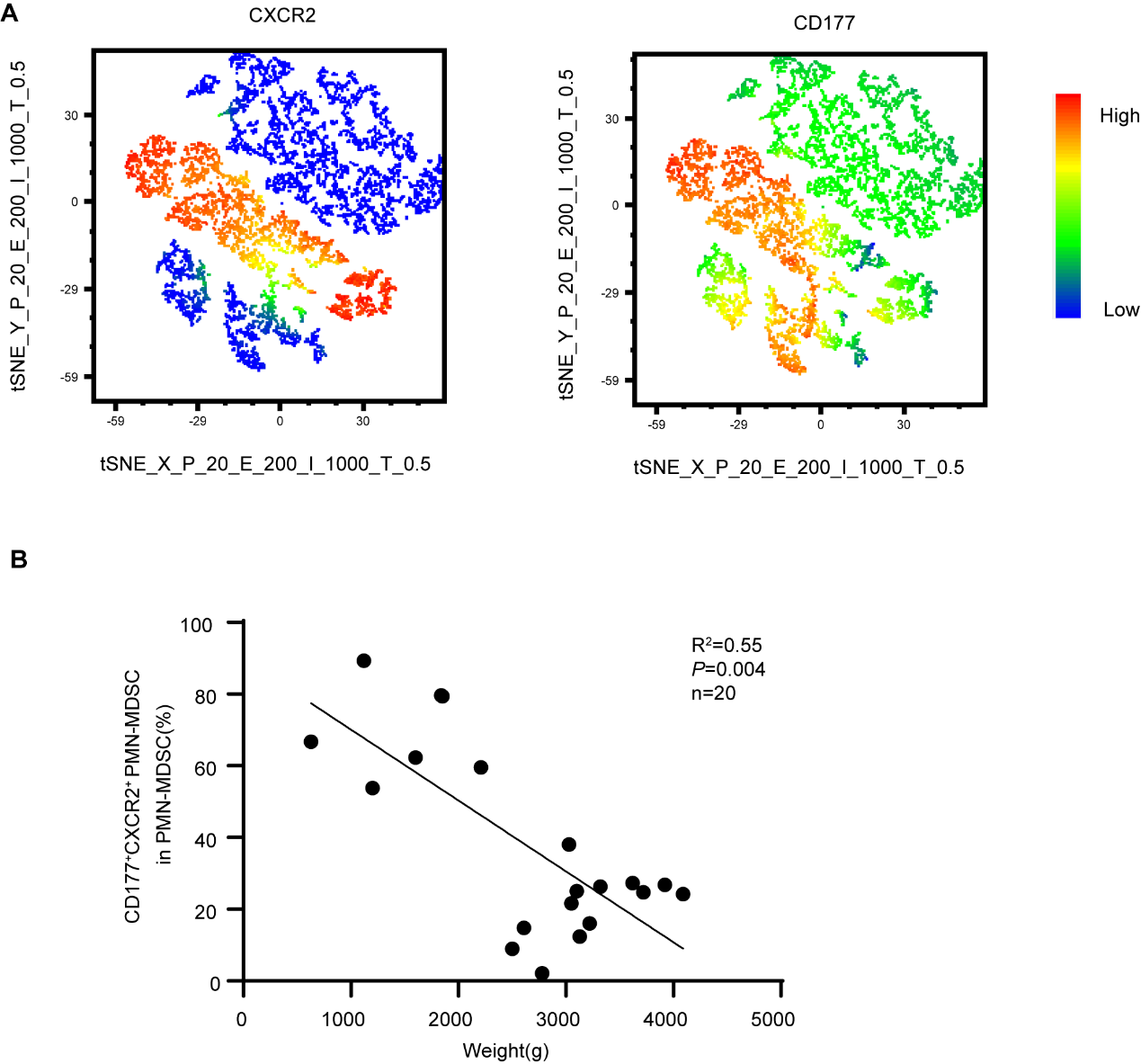


**Supplemental Figure2.**Analysis of PMN-MDSC subpopulations by flow cytometry, related to Figure2. **(A)** Pseudocolor tSNE plots of CD177 and CXCR2 expression. **(B)** Correlation between abundance of PMN C3 and body weight of infants. Spearman’s correlation coefficient was calculated, and actual P values are shown.
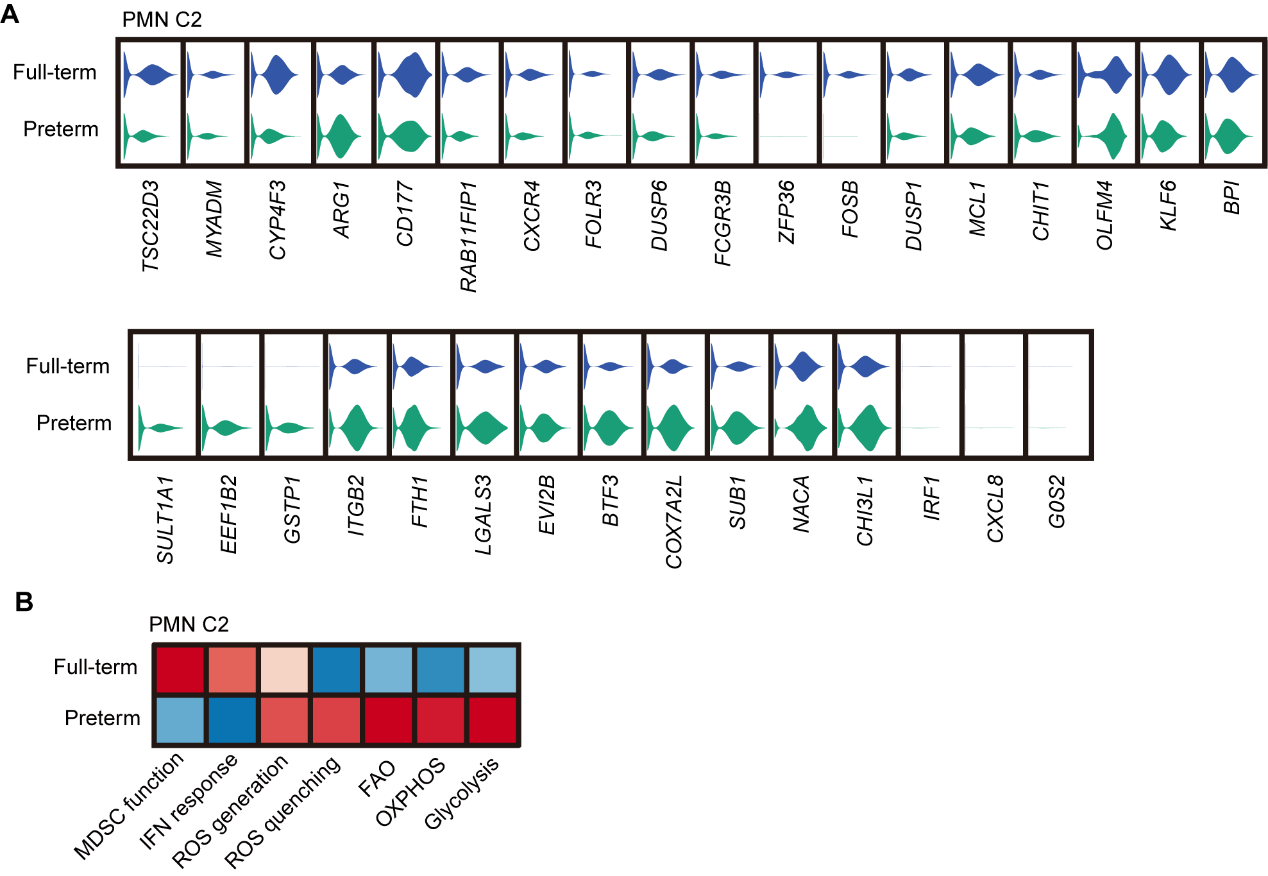


**Supplementary Figure 3.** Differential expression analysis of PMN C2 from full-term and preterm infants **(A)** Violin plots comparing gene expressions in PMN C2 between full-term and preterm infants. **(B)** Heatmap displaying normalized scores of molecular signatures in PMN C2 across full-term and preterm.

## Supplementary Table

**Supplementary Table 1.** **genes used for each molecular signatures for Figure1D**

| **Signature** | **Genes** |
| --- | --- |
| **Glycolysis** | *ACSS1, ACSS2, ADH1A, ADH1B, ADH1C, ADH4, ADH5, ADH6, ADH7, AKR1A1, ALDH1A3, ALDH1B1, ALDH2, ALDH3A1, ALDH3A2, ALDH3B1, ALDH3B2, ALDH7A1, ALDH9A1, ALDOA, ALDOB, ALDOC, BPGM, DLAT, DLD, ENO1, ENO2, ENO3, FBP1, FBP2, G6PC, G6PC2, GALM, GAPDH, GCK, GPI, HK1, HK2, HK3, LDHA, LDHAL6A, LDHAL6B, LDHB, LDHC, PCK1, PCK2, PDHA1, PDHA2, PDHB, PFKL, PFKM, PFKP, PGAM1, PGAM2, PGAM4, PGK1, PGK2, PGM1, PGM2, PKLR, PKM2, TPI1* |
| **OXPHOS** | *ATP12A, ATP4A, ATP4B, ATP5A1, ATP5B, ATP5C1, ATP5D, ATP5E, ATP5F1, ATP5G1, ATP5G1P5, ATP5G2, ATP5G3, ATP5H, ATP5I, ATP5J, ATP5J2, ATP5L, ATP5O, ATP6, ATP6AP1, ATP6V0A1, ATP6V0A2, ATP6V0A4, ATP6V0B, ATP6V0C, ATP6V0D1, ATP6V0D2, ATP6V0E1, ATP6V0E2, ATP6V1A, ATP6V1B1, ATP6V1B2, ATP6V1C1, ATP6V1C2, ATP6V1D, ATP6V1E1, ATP6V1E2, ATP6V1F, ATP6V1G1, ATP6V1G2, ATP6V1G3, ATP6V1H, ATP8, COX1, COX10, COX11, COX15, COX17, COX2, COX3, COX4I1, COX4I2, COX5A, COX5B, COX6A1, COX6A2, COX6B1, COX6B2, COX6C, COX6CP3, COX7A1, COX7A2, COX7A2L, COX7B, COX7B2, COX7C, COX8A, COX8C, CYC1, CYTB, LHPP, LOC100133737, LOC642502, LOC644310, LOC727947, ND1, ND2, ND3, ND4, ND4L, ND5, ND6, NDUFA1, NDUFA10, NDUFA11, NDUFA2, NDUFA3, NDUFA4, NDUFA4L2, NDUFA5, NDUFA6, NDUFA7, NDUFA8, NDUFA9, NDUFAB1, NDUFB1, NDUFB10, NDUFB2, NDUFB3, NDUFB4, NDUFB5, NDUFB6, NDUFB7, NDUFB8, NDUFB9, NDUFC1, NDUFC2, NDUFS1, NDUFS2, NDUFS3, NDUFS4, NDUFS5, NDUFS6, NDUFS7, NDUFS8, NDUFV1, NDUFV2, NDUFV3, PPA1, PPA2, SDHA, SDHB, SDHC, SDHD, TCIRG1, UQCR10, UQCR11, UQCRB, UQCRC1, UQCRC2, UQCRFS1, UQCRH, UQCRHL, UQCRQ* |
| **FAO** | *ABCD1, ABCD2, ABCD3, ACAA1, ACAD10, ACAD11, ACAD8, ACAD9, ACADL, ACADM, ACADS, ACADSB, ACADVL, ACOT8, ACOX1, ACOX2, ACOX3, ACOXL, ADIPOQ, AMACR, BDH2, CPT1A, CPT1B, CPT1C, CPT2, CRAT, CROT, DECR1, ECH1, ECHS1, ECI1, ECI2, EHHADH, ETFA, ETFB, ETFDH, GCDH, HADH, HADHA, HADHB, HSD17B4, IVD, LEP, NUDT19, PEX2, PEX7, PPARD, SCP2, SESN2, SLC25A17, SLC27A2* |
| **Stemness** | *CHD6, SUZ12, DEK, SMARCA5, CTCF, CBX3, CRTC2, ASRGL1, SLPI, EXOSC9, FUT4,  HCFC1, HMGN2, HMGB2, HNRNPA1, SRSF3, POLE4, PSMB7* |
| **ROS generation** | *MPO, NDUFA6, NDUFB4, NDUFS2, NQO1, CYBB, CYBA, NCF1, NCF2, NCF4* |
| **ROS quenching** | *ATOX1, CAT, G6PD, GCLC, GCLM, GLRX, GLRX2, GPX3, GPX4, GSR, MGST1, MSRA, PRDX1, PRDX2, SOD1, SOD2, TXNRD1, TXNRD2, PRDX4, PRDX6, SRXN1, STK25, NFE2L2, KEAP1, PRDX4, PRDX6, SRXN1, STK25, NFE2L2, KEAP1* |
| **IFN response** | *IFITM2, IFITM1, IFITM3, DDX5, IL1B, DDX6, DDX21, IFIT3, ISG15, IFIT2, IFIT1, MX1, IFI6, ISG20, MX2, IFIT5, IRF1* |
| **MDSC function** | *LTF, ARG1, STAT3, NOS1, NOS2, S100A9, S100A8, DEFA3, DEFA4, LTF, CAMP, LCN2, PGLYRP1, ORM1, MMP9, FCGR3B, CRISP3, CD24, S100P, CD177, S100A12, CEACAM8, MMP8, AZU1, MPO, HP, RETN* |
| **Migration** | *CXCR2, CXCR1, CXCL8* |
| **Proliferation** | *STMN1, MKI67, PCNA, TUBA1B, PCLAF, BIRC5* |

**Supplementary Table 2.** **The clinical characteristics of samples.**

| **Characteristic** | **Term** | **Preterm** | **Adult** |
| --- | --- | --- | --- |
| No. of subjects | 16 | 10 | 6 |
| Age(days) | 2.70±2.11 | 3.40±1.81 | 25.17±0.75(years) |
| Gender(M:F) | 1:1 | 3:2 | 1:2 |
| Body weight(kg) | 3.12±0.40 | 1.44±0.49 | 56.67±12.09 |
| Gestational age(week) | 38.31±1.14 | 29.90±2.60 |  |
| Antenatal steroids,n(%) | 0(0%) | 5(50%) |  |
| **Other diseases** |  | | |
| Feeding intolerance, n (%) | 0(0%) | 0(0%) |  |
| Respiratory distress syndrome,  n (%) | 0(0%) | 7(70%) |  |
| Neonatal infection, n (%) | 0(0%) | 0(0%) |  |
| Neonatal anemia, n (%) | 1(6.25%) | 2(20%) |  |
| Autoimmune diseases, n (%) | 0(0%) | 0(0%) |  |

**Supplementary Table 3.** **genes associated with the enriched pathways identified through GO analysis for Figure 3B**

| **ID** | **Description** | **pvalue** | **p.adjust** | **qvalue** | **geneID** |
| --- | --- | --- | --- | --- | --- |
| GO:0010955 | negative regulation of protein processing | 1.19E-05 | 0.006089 | 0.004521 | CST7/IL1R2/THBS1 |
| GO:0006639 | acylglycerol metabolic process | 4.34E-05 | 0.011453 | 0.008504 | MBOAT7/SORL1/PGS1/ACSL4 |
| GO:0006638 | neutral lipid metabolic process | 4.47E-05 | 0.011453 | 0.008504 | MBOAT7/SORL1/PGS1/ACSL4 |
| GO:0002604 | regulation of dendritic cell antigen processing and presentation | 0.000126 | 0.021924 | 0.016279 | SLC11A1/THBS1 |
| GO:0045861 | negative regulation of proteolysis | 0.000185 | 0.023644 | 0.017555 | CST7/SORL1/IL1R2/NAIP/THBS1 |
| GO:0046486 | glycerolipid metabolic process | 0.000308 | 0.029222 | 0.021697 | FAM126B/MBOAT7/SORL1/PGS1/ACSL4 |
| GO:2000117 | negative regulation of cysteine-type endopeptidase activity | 0.000314 | 0.029222 | 0.021697 | CST7/NAIP/THBS1 |
| GO:0002274 | myeloid leukocyte activation | 0.000368 | 0.031454 | 0.023354 | CST7/SLC11A1/STXBP2/THBS1 |
| GO:0002577 | regulation of antigen processing and presentation | 0.000433 | 0.031977 | 0.023743 | SLC11A1/THBS1 |
| GO:0006641 | triglyceride metabolic process | 0.000488 | 0.031977 | 0.023743 | MBOAT7/SORL1/ACSL4 |
| GO:0034755 | iron ion transmembrane transport | 0.000525 | 0.031977 | 0.023743 | SLC11A1/SLC25A37 |
| GO:0045017 | glycerolipid biosynthetic process | 0.000584 | 0.031977 | 0.023743 | FAM126B/MBOAT7/PGS1/ACSL4 |
| GO:0008654 | phospholipid biosynthetic process | 0.000593 | 0.031977 | 0.023743 | FAM126B/MBOAT7/IDI1/PGS1 |
| GO:0070861 | regulation of protein exit from endoplasmic reticulum | 0.000794 | 0.038744 | 0.028767 | UBE2J1/SORL1 |
| GO:0051604 | protein maturation | 0.001038 | 0.048362 | 0.035909 | CST7/SORL1/IL1R2/THBS1 |

**Supplementary Table 4.** **genes associated with the enriched pathways identified through GO analysis for Figure 3C**

| **ID** | **Description** | **pvalue** | **p.adjust** | **qvalue** | **geneID** |
| --- | --- | --- | --- | --- | --- |
| GO:0042742 | defense response to bacterium | 6.28E-08 | 3.47E-05 | 1.95E-05 | BPI/LCN2/CAMP/HP/LYZ/PGLYRP1 |
| GO:0032496 | response to lipopolysaccharide | 2.29E-06 | 0.000556 | 0.000312 | BPI/LCN2/CAMP/FOS/TRIB1 |
| GO:0002237 | response to molecule of bacterial origin | 3.02E-06 | 0.000556 | 0.000312 | BPI/LCN2/CAMP/FOS/TRIB1 |
| GO:0006979 | response to oxidative stress | 8.24E-06 | 0.000883 | 0.000496 | LCN2/HP/FOS/MCL1/DUSP1 |
| GO:0071222 | cellular response to lipopolysaccharide | 9.97E-06 | 0.000883 | 0.000496 | BPI/LCN2/CAMP/TRIB1 |
| GO:0071219 | cellular response to molecule of bacterial origin | 1.24E-05 | 0.000883 | 0.000496 | BPI/LCN2/CAMP/TRIB1 |
| GO:0000302 | response to reactive oxygen species | 1.26E-05 | 0.000883 | 0.000496 | LCN2/HP/FOS/DUSP1 |
| GO:0031640 | killing of cells of other organism | 1.28E-05 | 0.000883 | 0.000496 | CAMP/LYZ/PGLYRP1 |
| GO:0050829 | defense response to Gram-negative bacterium | 2.77E-05 | 0.001534 | 0.000861 | BPI/CAMP/LYZ |
